# 3-[(8-But­oxy­quinolin-2-yl)meth­yl]-1-(pyridin-2-ylmeth­yl)-1*H*-imidazol-3-ium hexa­fluoridophosphate

**DOI:** 10.1107/S1600536812013414

**Published:** 2012-04-04

**Authors:** Ping Zhang, Jing Jing, Da-Bin Qin

**Affiliations:** a Chemical Synthesis and Pollution Control Key Laboratory of Sichuan Province, School of Chemistry and Chemical Engineering, China West Normal University, Nanchong 637002, People’s Republic of China

## Abstract

In the cation of the title compound, C_23_H_25_N_4_O^+^·PF_6_^−^, the imidazolium ring make dihedral angles of 87.20 (6) and 79.89 (5)° with the pyridine ring and the quinoline system, respectively. In the crystal, C—H⋯F and C—H⋯N hydrogen bonds are observed.

## Related literature

For the first stable *N*-heterocyclic carbene, see: Arduengo *et al.* (1991 ▶). For the synthesis of 8-but­oxy-quinoline-2-carbaldehyde, see: Maffeo *et al.* (2003 ▶), of 8-but­oxy-2-chloro­methyl-quinoline, see: Fowelin *et al.* (2007 ▶) and of 2-((1*H*-imidazol-1-yl)meth­yl)pyridine, see: Chiu *et al.* (2005 ▶). For ionic liquids from imidazolium salts, see: Heller *et al.* (2010 ▶). For standard bond lengths, see: Allen *et al.* (1987 ▶).
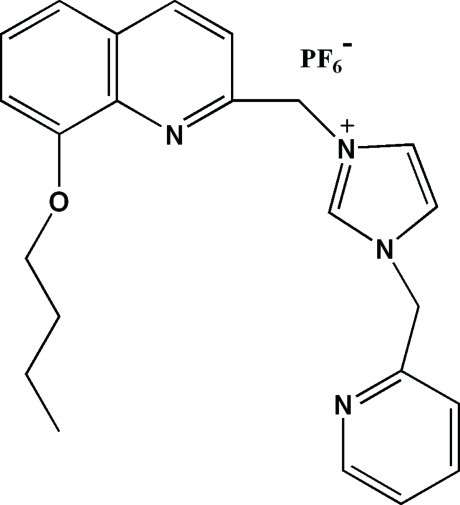


## Experimental

### 

#### Crystal data

C_23_H_25_N_4_O^+^·PF_6_^−^
*M* *_r_* = 518.44
Triclinic, 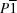

*a* = 9.975 (3) Å
*b* = 11.056 (4) Å
*c* = 12.382 (4) Å
α = 99.010 (4)°
β = 103.527 (3)°
γ = 112.734 (3)°
*V* = 1177.1 (7) Å^3^
*Z* = 2
Mo *K*α radiation
μ = 0.19 mm^−1^
*T* = 113 K
0.24 × 0.20 × 0.18 mm

#### Data collection

Rigaku Saturn CCD area-detector diffractometer
Absorption correction: multi-scan (*CrystalClear*; Rigaku, 2005 ▶) *T*_min_ = 0.956, *T*_max_ = 0.967
12352 measured reflections
5531 independent reflections
2844 reflections with *I* > 2σ(*I*)
*R*_int_ = 0.037

#### Refinement

*R*[*F* ^2^ > 2σ(*F* ^2^)] = 0.038
*wR*(*F* ^2^) = 0.087
*S* = 1.01
5531 reflections
317 parameters
H-atom parameters constrained
Δρ_max_ = 0.58 e Å^−3^
Δρ_min_ = −0.24 e Å^−3^

### 

Data collection: *CrystalClear* (Rigaku, 2005 ▶); cell refinement: *CrystalClear*; data reduction: *CrystalClear*; program(s) used to solve structure: *SHELXS97* (Sheldrick, 2008 ▶); program(s) used to refine structure: *SHELXL97* (Sheldrick, 2008 ▶); molecular graphics: *SHELXTL* (Sheldrick, 2008 ▶); software used to prepare material for publication: *CrystalStructure* (Rigaku, 2005 ▶).

## Supplementary Material

Crystal structure: contains datablock(s) global, I. DOI: 10.1107/S1600536812013414/fj2531sup1.cif

Structure factors: contains datablock(s) I. DOI: 10.1107/S1600536812013414/fj2531Isup2.hkl

Supplementary material file. DOI: 10.1107/S1600536812013414/fj2531Isup3.cml

Additional supplementary materials:  crystallographic information; 3D view; checkCIF report

## Figures and Tables

**Table 1:** Hydrogen-bond geometry (Å, °)

*D*—H⋯*A*	*D*—H	H⋯*A*	*D*⋯*A*	*D*—H⋯*A*
C4—H4⋯N4^i^	0.95	2.58	3.343 (2)	138
C14—H14*B*⋯F1^ii^	0.99	2.50	3.425 (2)	156
C16—H16⋯F4^i^	0.95	2.46	3.361 (2)	159
C17—H17⋯F6^ii^	0.95	2.32	3.223 (2)	158
C23—H23⋯F4^iii^	0.95	2.52	3.355 (2)	147

Symmetry codes: (i) 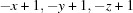; (ii) 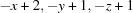; (iii) 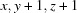.

**Fig. 1.**
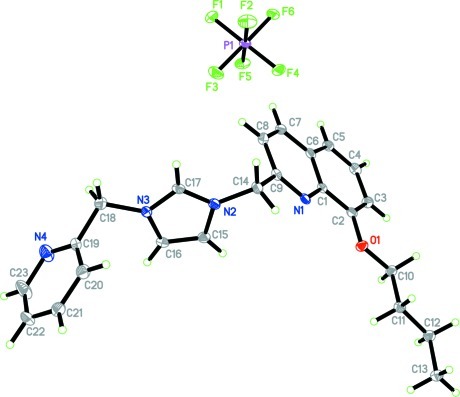
A view of the molecular structure of the title compound with atom numbering. The displacement ellipsoids are drawn at the 30% probability level.

**Fig. 2.**
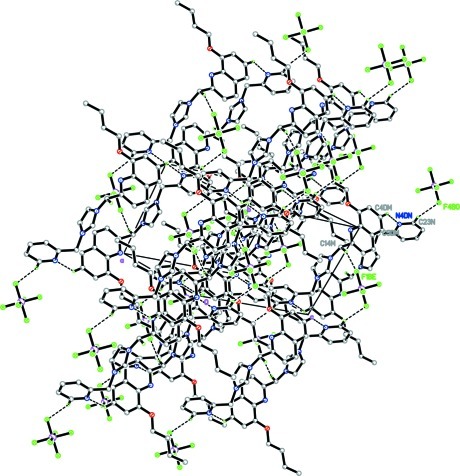
The crystal packing of the title compound, showing the cations and anions linked via C—H···F interactions (dashed lines). H atoms not involved in these interactions have been omitted for clarity. [symmetry codes: (i) 1 - x,1 - y,1 - z; (ii) 2 - x,1 - y,1 - z; (iii) x,1 + y,1 + z].

## supplementary crystallographic information

### Comment

since the isolation of the first stable derivative of N-heterocyclic carbenes (Arduengo *et al.*, 1991), the chemistry of N-heterocyclic carbenes has been studied intensely, so numerous stable NHC ligands has been prepared. Imidazolium salts are considerable good precursor for the synthesis of N-heterocyclic carbenes. In addition, the study of ionic liquids of imidazolium salts (Heller *et al.*, 2010) have been reported during these years.

We report herein the synthesis and crystal structure of the title compound.

The molecular structure of the title compound is shown in Fig. 1. Bond lengths (Allen *et al.*, 1987) and angles in the cation are normal. The imidazolium ring make dihedral angles with the pyridine ring and the quinoline ring of 87.20 (6)° and 79.89 (5)°, respectively. In the crystal there are weak π···π interactions involving the pyridine ring with centroid-centroid distances, *Cg*2···*Cg*4^i^ of 3.692 (9). [symmetry codes: (i) 1 - *x*, 2 - *y*, 1 - *z*] In addition, extensive C—H···F and C—H···N hydrogen bonds which contribute to the stability of molecular structure are observed (Table 1 and Fig. 2).

### Experimental

8-Butoxy-2-chloromethyl-quinoline (Maffeo *et al.*, 2003 and Fowelin *et al.*, 2007) (2.49 g, 10 mmol) was added to a solution of 2-((1*H*-imidazol-1-yl)methyl)pyridine (Chiu *et al.*, 2005) (1.59 g, 10 mmol) in 50 ml of THF. The mixture was refluxed for 48 h. The resulting precipitate was isolated and washed with THF(2 × 5 ml) and was then dissolved in methanol (20 ml). Adding an excess of NH_4_PF_6_ to the aqueous solution yielded the title compound. Colorless single crystals suitable for X-ray diffraction were obtained by recrystallization from acetonitrile and diethyl ether(*v*/*v*=1/4).

### Refinement

H atoms were placed in calculated positions with C—H = 0.95–0.99 Å, and refined in riding mode with *U*_iso_(H) = 1.2*U*_eq_(C).

### Crystal data

**Table:**

C_23_H_25_N_4_O^+^·PF_6_^−^	*Z* = 2
*M**_r_* = 518.44	*F*(000) = 536
Triclinic, *P*1	*D*_x_ = 1.463 Mg m^−^^3^
Hall symbol: -P 1	Mo *K*α radiation, λ = 0.71073 Å
*a* = 9.975 (3) Å	Cell parameters from 3783 reflections
*b* = 11.056 (4) Å	θ = 1.8–27.9°
*c* = 12.382 (4) Å	µ = 0.19 mm^−^^1^
α = 99.010 (4)°	*T* = 113 K
β = 103.527 (3)°	Prism, colourless
γ = 112.734 (3)°	0.24 × 0.20 × 0.18 mm
*V* = 1177.1 (7) Å^3^	

### Data collection

**Table:**

Rigaku Saturn CCD area-detector diffractometer	5531 independent reflections
Radiation source: rotating anode	2844 reflections with *I* > 2σ(*I*)
Multilayer monochromator	*R*_int_ = 0.037
Detector resolution: 14.63 pixels mm^-1^	θ_max_ = 27.9°, θ_min_ = 1.8°
ω and φ scans	*h* = −13→13
Absorption correction: multi-scan (*CrystalClear*; Rigaku, 2005)	*k* = −13→14
*T*_min_ = 0.956, *T*_max_ = 0.967	*l* = −15→16
12352 measured reflections	

### Refinement

**Table:**

Refinement on *F*^2^	Primary atom site location: structure-invariant direct methods
Least-squares matrix: full	Secondary atom site location: difference Fourier map
*R*[*F*^2^ > 2σ(*F*^2^)] = 0.038	Hydrogen site location: inferred from neighbouring sites
*wR*(*F*^2^) = 0.087	H-atom parameters constrained
*S* = 1.01	*w* = 1/[σ^2^(*F*_o_^2^) + (0.0259*P*)^2^] where *P* = (*F*_o_^2^ + 2*F*_c_^2^)/3
5531 reflections	(Δ/σ)_max_ = 0.001
317 parameters	Δρ_max_ = 0.58 e Å^−^^3^
0 restraints	Δρ_min_ = −0.24 e Å^−^^3^

### Special details

**Table:**

Geometry. All e.s.d.'s (except the e.s.d. in the dihedral angle between two l.s. planes) are estimated using the full covariance matrix. The cell e.s.d.'s are taken into account individually in the estimation of e.s.d.'s in distances, angles and torsion angles; correlations between e.s.d.'s in cell parameters are only used when they are defined by crystal symmetry. An approximate (isotropic) treatment of cell e.s.d.'s is used for estimating e.s.d.'s involving l.s. planes.
Refinement. Refinement of *F*^2^ against ALL reflections. The weighted *R*-factor *wR* and goodness of fit *S* are based on *F*^2^, conventional *R*-factors *R* are based on *F*, with *F* set to zero for negative *F*^2^. The threshold expression of *F*^2^ > σ(*F*^2^) is used only for calculating *R*-factors(gt) *etc*. and is not relevant to the choice of reflections for refinement. *R*-factors based on *F*^2^ are statistically about twice as large as those based on *F*, and *R*- factors based on ALL data will be even larger.

### Fractional atomic coordinates and isotropic or equivalent isotropic displacement parameters (Å^2^)

**Table:**

	*x*	*y*	*z*	*U*_iso_*/*U*_eq_	
O1	0.18574 (14)	−0.05015 (11)	0.50857 (9)	0.0261 (3)	
N1	0.46828 (17)	0.15399 (13)	0.59818 (11)	0.0207 (3)	
N2	0.67464 (17)	0.44123 (14)	0.81155 (11)	0.0222 (3)	
N3	0.76044 (18)	0.65447 (14)	0.89753 (11)	0.0243 (3)	
N4	0.7840 (2)	0.96366 (16)	1.02355 (13)	0.0430 (5)	
C1	0.4087 (2)	0.10204 (16)	0.48018 (13)	0.0199 (4)	
C2	0.2576 (2)	−0.00743 (17)	0.43121 (14)	0.0230 (4)	
C3	0.1955 (2)	−0.06110 (18)	0.31350 (14)	0.0266 (4)	
H3	0.0950	−0.1341	0.2805	0.032*	
C4	0.2801 (2)	−0.00833 (18)	0.24126 (14)	0.0272 (4)	
H4	0.2356	−0.0472	0.1600	0.033*	
C5	0.4231 (2)	0.09621 (17)	0.28470 (14)	0.0258 (4)	
H5	0.4769	0.1310	0.2341	0.031*	
C6	0.4921 (2)	0.15361 (17)	0.40557 (14)	0.0229 (4)	
C7	0.6411 (2)	0.26183 (17)	0.45754 (15)	0.0266 (4)	
H7	0.7005	0.2993	0.4109	0.032*	
C8	0.6998 (2)	0.31251 (17)	0.57488 (15)	0.0275 (4)	
H8	0.8000	0.3854	0.6107	0.033*	
C9	0.6087 (2)	0.25439 (17)	0.64230 (14)	0.0233 (4)	
C10	0.0333 (2)	−0.15994 (17)	0.46166 (14)	0.0256 (4)	
H10A	0.0348	−0.2408	0.4154	0.031*	
H10B	−0.0352	−0.1324	0.4105	0.031*	
C11	−0.0249 (2)	−0.19441 (17)	0.55992 (14)	0.0263 (4)	
H11A	−0.0195	−0.1114	0.6084	0.032*	
H11B	0.0421	−0.2253	0.6087	0.032*	
C12	−0.1897 (2)	−0.30559 (18)	0.51700 (14)	0.0288 (4)	
H12A	−0.2554	−0.2771	0.4642	0.035*	
H12B	−0.1937	−0.3902	0.4725	0.035*	
C13	−0.2529 (2)	−0.33511 (18)	0.61483 (15)	0.0319 (5)	
H13A	−0.1896	−0.3654	0.6664	0.048*	
H13B	−0.3588	−0.4069	0.5831	0.048*	
H13C	−0.2512	−0.2521	0.6583	0.048*	
C14	0.6703 (2)	0.30680 (16)	0.77246 (14)	0.0252 (4)	
H14A	0.6045	0.2414	0.8059	0.030*	
H14B	0.7753	0.3138	0.8003	0.030*	
C15	0.5510 (2)	0.47198 (18)	0.79151 (14)	0.0265 (4)	
H15	0.4474	0.4105	0.7479	0.032*	
C16	0.6041 (2)	0.60457 (18)	0.84485 (14)	0.0267 (4)	
H16	0.5454	0.6547	0.8462	0.032*	
C17	0.7995 (2)	0.55370 (17)	0.87609 (13)	0.0237 (4)	
H17	0.9003	0.5608	0.9026	0.028*	
C18	0.8687 (2)	0.79717 (18)	0.96022 (16)	0.0376 (5)	
H18A	0.8857	0.8516	0.9042	0.045*	
H18B	0.9686	0.8015	1.0025	0.045*	
C19	0.8087 (2)	0.85781 (17)	1.04502 (15)	0.0274 (4)	
C20	0.7801 (2)	0.80741 (19)	1.13636 (15)	0.0317 (5)	
H20	0.7968	0.7308	1.1481	0.038*	
C21	0.7279 (2)	0.86810 (18)	1.20933 (15)	0.0320 (5)	
H21	0.7099	0.8355	1.2736	0.038*	
C22	0.7014 (2)	0.97635 (19)	1.18994 (16)	0.0366 (5)	
H22	0.6644	1.0200	1.2399	0.044*	
C23	0.7295 (3)	1.0200 (2)	1.09668 (18)	0.0528 (7)	
H23	0.7096	1.0944	1.0825	0.063*	
P1	0.80855 (6)	0.43611 (5)	0.15376 (4)	0.02658 (13)	
F1	0.93828 (12)	0.57111 (10)	0.14046 (9)	0.0424 (3)	
F2	0.93902 (13)	0.42366 (12)	0.24704 (9)	0.0496 (3)	
F3	0.78373 (14)	0.52848 (11)	0.25236 (8)	0.0464 (3)	
F4	0.68142 (13)	0.30267 (10)	0.16650 (9)	0.0427 (3)	
F5	0.68041 (12)	0.45015 (10)	0.05947 (8)	0.0379 (3)	
F6	0.83460 (13)	0.34588 (10)	0.05310 (9)	0.0395 (3)	

### Atomic displacement parameters (Å^2^)

**Table:**

	*U*^11^	*U*^22^	*U*^33^	*U*^12^	*U*^13^	*U*^23^
O1	0.0258 (8)	0.0272 (7)	0.0218 (6)	0.0064 (6)	0.0117 (6)	0.0048 (5)
N1	0.0231 (9)	0.0193 (8)	0.0218 (8)	0.0122 (7)	0.0083 (7)	0.0022 (6)
N2	0.0215 (9)	0.0244 (8)	0.0206 (8)	0.0109 (7)	0.0075 (7)	0.0034 (7)
N3	0.0233 (9)	0.0214 (8)	0.0245 (8)	0.0071 (7)	0.0104 (7)	−0.0007 (7)
N4	0.0672 (14)	0.0345 (10)	0.0355 (10)	0.0252 (10)	0.0244 (10)	0.0121 (8)
C1	0.0242 (11)	0.0191 (9)	0.0205 (9)	0.0134 (8)	0.0083 (8)	0.0048 (8)
C2	0.0277 (11)	0.0234 (10)	0.0254 (10)	0.0151 (9)	0.0135 (9)	0.0089 (8)
C3	0.0263 (11)	0.0277 (10)	0.0235 (10)	0.0108 (9)	0.0073 (9)	0.0049 (8)
C4	0.0346 (12)	0.0320 (11)	0.0180 (10)	0.0186 (10)	0.0077 (9)	0.0060 (8)
C5	0.0356 (12)	0.0277 (10)	0.0244 (10)	0.0180 (10)	0.0173 (9)	0.0125 (8)
C6	0.0290 (11)	0.0213 (10)	0.0262 (10)	0.0162 (9)	0.0122 (9)	0.0080 (8)
C7	0.0308 (12)	0.0242 (10)	0.0340 (11)	0.0149 (9)	0.0191 (9)	0.0121 (9)
C8	0.0246 (11)	0.0208 (10)	0.0342 (11)	0.0085 (9)	0.0093 (9)	0.0045 (8)
C9	0.0281 (11)	0.0200 (9)	0.0240 (10)	0.0141 (9)	0.0079 (9)	0.0033 (8)
C10	0.0221 (11)	0.0237 (10)	0.0257 (10)	0.0057 (9)	0.0081 (9)	0.0035 (8)
C11	0.0292 (12)	0.0269 (10)	0.0257 (10)	0.0138 (9)	0.0120 (9)	0.0067 (8)
C12	0.0290 (12)	0.0283 (10)	0.0267 (10)	0.0091 (9)	0.0121 (9)	0.0056 (8)
C13	0.0351 (13)	0.0314 (11)	0.0372 (11)	0.0160 (10)	0.0203 (10)	0.0136 (9)
C14	0.0261 (11)	0.0226 (9)	0.0259 (10)	0.0119 (9)	0.0068 (9)	0.0033 (8)
C15	0.0181 (10)	0.0295 (11)	0.0293 (10)	0.0102 (9)	0.0063 (9)	0.0036 (9)
C16	0.0243 (11)	0.0305 (11)	0.0308 (11)	0.0154 (9)	0.0130 (9)	0.0078 (9)
C17	0.0212 (11)	0.0310 (10)	0.0193 (9)	0.0119 (9)	0.0085 (8)	0.0040 (8)
C18	0.0336 (13)	0.0292 (11)	0.0411 (12)	0.0054 (10)	0.0185 (10)	−0.0015 (9)
C19	0.0282 (12)	0.0216 (10)	0.0280 (10)	0.0082 (9)	0.0103 (9)	0.0007 (8)
C20	0.0339 (13)	0.0289 (11)	0.0357 (11)	0.0158 (10)	0.0114 (10)	0.0125 (9)
C21	0.0320 (12)	0.0333 (11)	0.0232 (10)	0.0074 (10)	0.0092 (9)	0.0063 (9)
C22	0.0427 (14)	0.0368 (12)	0.0326 (11)	0.0203 (11)	0.0174 (10)	0.0002 (10)
C23	0.090 (2)	0.0404 (13)	0.0560 (15)	0.0466 (14)	0.0362 (14)	0.0196 (12)
P1	0.0242 (3)	0.0309 (3)	0.0284 (3)	0.0139 (2)	0.0126 (2)	0.0076 (2)
F1	0.0294 (7)	0.0331 (6)	0.0621 (8)	0.0080 (6)	0.0193 (6)	0.0145 (6)
F2	0.0400 (8)	0.0768 (9)	0.0428 (7)	0.0351 (7)	0.0096 (6)	0.0261 (7)
F3	0.0481 (8)	0.0562 (8)	0.0369 (7)	0.0280 (7)	0.0181 (6)	−0.0014 (6)
F4	0.0401 (8)	0.0363 (6)	0.0662 (8)	0.0170 (6)	0.0334 (6)	0.0272 (6)
F5	0.0286 (7)	0.0558 (7)	0.0359 (6)	0.0209 (6)	0.0123 (5)	0.0211 (6)
F6	0.0346 (7)	0.0393 (6)	0.0431 (6)	0.0149 (6)	0.0204 (6)	−0.0012 (5)

### Geometric parameters (Å, º)

**Table:**

O1—C2	1.3621 (19)	C11—H11A	0.9900
O1—C10	1.436 (2)	C11—H11B	0.9900
N1—C9	1.320 (2)	C12—C13	1.514 (2)
N1—C1	1.3729 (19)	C12—H12A	0.9900
N2—C17	1.327 (2)	C12—H12B	0.9900
N2—C15	1.380 (2)	C13—H13A	0.9800
N2—C14	1.470 (2)	C13—H13B	0.9800
N3—C17	1.324 (2)	C13—H13C	0.9800
N3—C16	1.381 (2)	C14—H14A	0.9900
N3—C18	1.471 (2)	C14—H14B	0.9900
N4—C19	1.338 (2)	C15—C16	1.341 (2)
N4—C23	1.348 (2)	C15—H15	0.9500
C1—C6	1.425 (2)	C16—H16	0.9500
C1—C2	1.427 (2)	C17—H17	0.9500
C2—C3	1.372 (2)	C18—C19	1.515 (2)
C3—C4	1.410 (2)	C18—H18A	0.9900
C3—H3	0.9500	C18—H18B	0.9900
C4—C5	1.353 (2)	C19—C20	1.377 (2)
C4—H4	0.9500	C20—C21	1.355 (2)
C5—C6	1.414 (2)	C20—H20	0.9500
C5—H5	0.9500	C21—C22	1.366 (2)
C6—C7	1.411 (2)	C21—H21	0.9500
C7—C8	1.364 (2)	C22—C23	1.366 (3)
C7—H7	0.9500	C22—H22	0.9500
C8—C9	1.417 (2)	C23—H23	0.9500
C8—H8	0.9500	P1—F4	1.5906 (12)
C9—C14	1.510 (2)	P1—F2	1.5911 (12)
C10—C11	1.506 (2)	P1—F5	1.5916 (11)
C10—H10A	0.9900	P1—F3	1.5941 (11)
C10—H10B	0.9900	P1—F6	1.6058 (11)
C11—C12	1.524 (2)	P1—F1	1.6165 (12)
			
C2—O1—C10	116.58 (13)	H13A—C13—H13B	109.5
C9—N1—C1	117.78 (15)	C12—C13—H13C	109.5
C17—N2—C15	108.25 (14)	H13A—C13—H13C	109.5
C17—N2—C14	125.14 (15)	H13B—C13—H13C	109.5
C15—N2—C14	126.58 (15)	N2—C14—C9	111.12 (13)
C17—N3—C16	108.55 (15)	N2—C14—H14A	109.4
C17—N3—C18	125.01 (17)	C9—C14—H14A	109.4
C16—N3—C18	126.31 (15)	N2—C14—H14B	109.4
C19—N4—C23	116.48 (16)	C9—C14—H14B	109.4
N1—C1—C6	122.35 (16)	H14A—C14—H14B	108.0
N1—C1—C2	118.46 (15)	C16—C15—N2	107.39 (16)
C6—C1—C2	119.20 (15)	C16—C15—H15	126.3
O1—C2—C3	125.06 (17)	N2—C15—H15	126.3
O1—C2—C1	115.38 (15)	C15—C16—N3	106.97 (16)
C3—C2—C1	119.56 (16)	C15—C16—H16	126.5
C2—C3—C4	120.29 (18)	N3—C16—H16	126.5
C2—C3—H3	119.9	N3—C17—N2	108.84 (16)
C4—C3—H3	119.9	N3—C17—H17	125.6
C5—C4—C3	121.72 (17)	N2—C17—H17	125.6
C5—C4—H4	119.1	N3—C18—C19	111.41 (15)
C3—C4—H4	119.1	N3—C18—H18A	109.3
C4—C5—C6	119.87 (17)	C19—C18—H18A	109.3
C4—C5—H5	120.1	N3—C18—H18B	109.3
C6—C5—H5	120.1	C19—C18—H18B	109.3
C7—C6—C5	123.27 (16)	H18A—C18—H18B	108.0
C7—C6—C1	117.37 (16)	N4—C19—C20	122.50 (17)
C5—C6—C1	119.36 (17)	N4—C19—C18	114.66 (16)
C8—C7—C6	119.95 (17)	C20—C19—C18	122.83 (16)
C8—C7—H7	120.0	C21—C20—C19	119.39 (17)
C6—C7—H7	120.0	C21—C20—H20	120.3
C7—C8—C9	118.76 (17)	C19—C20—H20	120.3
C7—C8—H8	120.6	C20—C21—C22	119.65 (17)
C9—C8—H8	120.6	C20—C21—H21	120.2
N1—C9—C8	123.78 (16)	C22—C21—H21	120.2
N1—C9—C14	115.93 (16)	C21—C22—C23	118.07 (18)
C8—C9—C14	120.29 (17)	C21—C22—H22	121.0
O1—C10—C11	108.68 (13)	C23—C22—H22	121.0
O1—C10—H10A	110.0	N4—C23—C22	123.88 (18)
C11—C10—H10A	110.0	N4—C23—H23	118.1
O1—C10—H10B	110.0	C22—C23—H23	118.1
C11—C10—H10B	110.0	F4—P1—F2	90.58 (7)
H10A—C10—H10B	108.3	F4—P1—F5	90.40 (7)
C10—C11—C12	111.93 (14)	F2—P1—F5	178.92 (7)
C10—C11—H11A	109.2	F4—P1—F3	90.28 (7)
C12—C11—H11A	109.2	F2—P1—F3	90.96 (7)
C10—C11—H11B	109.2	F5—P1—F3	89.48 (6)
C12—C11—H11B	109.2	F4—P1—F6	90.85 (6)
H11A—C11—H11B	107.9	F2—P1—F6	89.68 (7)
C13—C12—C11	112.43 (15)	F5—P1—F6	89.86 (6)
C13—C12—H12A	109.1	F3—P1—F6	178.69 (6)
C11—C12—H12A	109.1	F4—P1—F1	179.61 (7)
C13—C12—H12B	109.1	F2—P1—F1	89.25 (7)
C11—C12—H12B	109.1	F5—P1—F1	89.76 (6)
H12A—C12—H12B	107.8	F3—P1—F1	90.07 (7)
C12—C13—H13A	109.5	F6—P1—F1	88.80 (6)
C12—C13—H13B	109.5		
			
C9—N1—C1—C6	−0.7 (2)	C10—C11—C12—C13	−176.49 (14)
C9—N1—C1—C2	179.40 (15)	C17—N2—C14—C9	−122.16 (17)
C10—O1—C2—C3	0.0 (2)	C15—N2—C14—C9	60.2 (2)
C10—O1—C2—C1	179.81 (13)	N1—C9—C14—N2	−106.79 (17)
N1—C1—C2—O1	0.2 (2)	C8—C9—C14—N2	73.1 (2)
C6—C1—C2—O1	−179.75 (13)	C17—N2—C15—C16	0.15 (19)
N1—C1—C2—C3	180.00 (14)	C14—N2—C15—C16	178.14 (14)
C6—C1—C2—C3	0.1 (2)	N2—C15—C16—N3	−0.05 (19)
O1—C2—C3—C4	179.65 (15)	C17—N3—C16—C15	−0.07 (19)
C1—C2—C3—C4	−0.2 (2)	C18—N3—C16—C15	175.97 (15)
C2—C3—C4—C5	−0.5 (3)	C16—N3—C17—N2	0.16 (19)
C3—C4—C5—C6	1.1 (3)	C18—N3—C17—N2	−175.93 (14)
C4—C5—C6—C7	179.41 (16)	C15—N2—C17—N3	−0.19 (19)
C4—C5—C6—C1	−1.2 (2)	C14—N2—C17—N3	−178.22 (13)
N1—C1—C6—C7	0.1 (2)	C17—N3—C18—C19	−137.01 (17)
C2—C1—C6—C7	−179.98 (14)	C16—N3—C18—C19	47.6 (2)
N1—C1—C6—C5	−179.33 (14)	C23—N4—C19—C20	0.2 (3)
C2—C1—C6—C5	0.6 (2)	C23—N4—C19—C18	179.58 (19)
C5—C6—C7—C8	179.60 (15)	N3—C18—C19—N4	−117.59 (19)
C1—C6—C7—C8	0.2 (2)	N3—C18—C19—C20	61.8 (2)
C6—C7—C8—C9	0.1 (2)	N4—C19—C20—C21	−1.4 (3)
C1—N1—C9—C8	1.0 (2)	C18—C19—C20—C21	179.30 (18)
C1—N1—C9—C14	−179.09 (13)	C19—C20—C21—C22	1.4 (3)
C7—C8—C9—N1	−0.7 (3)	C20—C21—C22—C23	−0.3 (3)
C7—C8—C9—C14	179.37 (15)	C19—N4—C23—C22	1.0 (4)
C2—O1—C10—C11	178.73 (13)	C21—C22—C23—N4	−0.9 (4)
O1—C10—C11—C12	177.10 (14)		

### Hydrogen-bond geometry (Å, º)

**Table:**

*D*—H···*A*	*D*—H	H···*A*	*D*···*A*	*D*—H···*A*
C4—H4···N4^i^	0.95	2.58	3.343 (2)	138
C14—H14*B*···F1^ii^	0.99	2.50	3.425 (2)	156
C16—H16···F4^i^	0.95	2.46	3.361 (2)	159
C17—H17···F6^ii^	0.95	2.32	3.223 (2)	158
C23—H23···F4^iii^	0.95	2.52	3.355 (2)	147

Symmetry codes: (i) −*x*+1, −*y*+1, −*z*+1; (ii) −*x*+2, −*y*+1, −*z*+1; (iii) *x*, *y*+1, *z*+1.
